# Potential blocker of SARS-CoV entry and a narrow functionality of its spike protein motifs on Qubevirus platform

**DOI:** 10.1016/j.jbc.2025.110371

**Published:** 2025-06-12

**Authors:** Aristide Dzelamonyuy, Augustin Ntemafack, Millie M. Georgiadis, Alain Bopda Waffo

**Affiliations:** Department of Biochemistry and Molecular Biology, Indiana University School of Medicine

**Keywords:** Qubevirus, A_1_ minor coat protein, SARS-CoV, spike protein, ACE2, chimeric epitope, LPETG, Biot-tag

## Abstract

Targeted disruption of SARS-CoV entry remains a critical strategy in antiviral therapeutic design. Central to this process is the viral spike (S) protein, which mediates host recognition *via* interactions with the human angiotensin-converting enzyme 2 (hACE2). Here, we expand our previous work by identifying the smallest active spike (S) protein binding motif (RBSM) and key residues of SARS-CoV (S_473–492_) that recognize hACE2. Using the Qubevirus (Qβ) platform, we validated five essential residues (L472, N473, N479, D480, and Y491) that are critical for SARS-CoV binding and entry. Qβ phage-displayed RBSM variants disrupted hACE2 recognition and infection initiation. An engineered RBSM insert containing all five mutant residues completely abolished recognition and binding to both hACE2 and anti-RBD antibodies. Furthermore, QβRBSM1 exhibits no cytotoxic effect on HEK293T cells and reduces the infectivity of SARS-CoV pseudovirus in a competitive assay, as a blocker of SARS-CoV entry. In addition, building upon our previous studies, we determined the optimal positioning of a chimera comprising the three epitopes mapped, fused with an LPTEG/Biot-tag at the N-terminus of the Qβ-A_1_ minor coat protein for anti-S antibody titration. We determined the optimal chimera tag configuration to be epitope 3 (S_781–800_) fused directly with the A_1_ at the N terminus, followed by epitope 1 (S_441–460_), epitope 2 (S_601–620_), and the tag at the C terminus. This work provides key insights into the druggability of the RBSM for developing SARS-CoV inhibitors and lays the foundation for designing a biosensor for antibody monitoring and a potential subunit vaccine.

The societal impact associated with severe acute respiratory syndrome (SARS) diseases is astronomical due to the significant morbidity, mortality, and high rate of transmission (1, 2, 3, 4, 5, 6). SARS disease is caused by two major coronaviruses referred to as SARS-CoV and SARS-CoV-2 (7, 8, 9, 10, 11). The first SARS outbreak was caused by SARS-CoV in Guangdong province in November 2002, and the second by SARS-CoV-2 in February 2020 in Wuhan, China (9, 12, 13, 14, 15). Unlike SARS-CoV, the incubation period of SARS-CoV-2 is often longer and can persist for up to 14 days (16). Ultimately, the incubation period is conditioned by tropism since both coronaviruses initiate infection through the recognition of the common receptor, the human angiotensin-converting enzyme 2 (hACE2), expressed by the host cell (17, 18, 19). The spike (S) protein of both SARS-CoVs is common with 85% sequence similarity and is essential for infection initiation and host immune system stimulation (20, 21). In addition to the S protein, the ∼ 30 kb positive sense RNA genome of both SARS-CoVs with similar organization encodes for the nuclear (N), membrane (M), and envelop (E) proteins (22, 23, 24). The structure, sequence, and domains of the S protein play a crucial role in disease severity; thus, it is druggable and a target for therapeutic agents' development. Similar to other human coronaviral diseases, SARS-CoV variants likely originate from zoonotic events (25) such as mutations of the spike (S) protein receptor-binding domain (RBD) determinants (26). To date, all the vaccines that have been approved for SARS-CoV-2 infection have been derived from the functional S protein determinants. Currently, there is no approved vaccine or drug against SARS-CoV. We recently found that the smallest active spike (S) protein binding motif (receptor-binding small motif, RBSM) of the SARS-CoV is made up of 55 amino acids and binds to the host receptor hACE2 (27). In addition, we mapped three major epitopes of the S protein using the established A_1_ minor coat of the Qubevirus (27).

Several studies indicate that the S protein is partially exposed on the SARS-CoVs platform and consists of ∼ 1255 amino acids (10, 28, 29). Of the four major structural proteins of SARS-CoV, the S is the most exposed and is characteristic of coronaviruses (30), with only 20 to 27% of amino acid sequence similarity among them (31). As a key element of infection, the difference in amino acid sequence is probably assigned to different functionalities. The S protein is a large glycoprotein anchored into the viral envelope. However, the number of N-glycosylation and O-glycosylation sites are distinct, and positions on the S of SARS-CoV are 10 and 2, respectively, and 9 and 3 on SARS-CoV-2, respectively (32, 33). The S protein comprises two domains, S1 and S2, which function as noncovalently bonded subunits (34). The S1 is situated between residues 14 and 641 and consists of two subdomains, S1a and S1b (35), and is predicted to be responsible for the virus binding to its host cell receptor, ACE2 (36, 37), which is now confirmed by our previous report. SARS-CoV binds to hACE2 through a putative binding fragment on S1b, which we identified using the RNA phage display system. Two heptad repeat region domains of the S2, the transmembrane subunit, are known to be HR1 and HR2 (38). During the infection cycle, both HRs facilitate viral and cellular membrane fusion in the fusogenic state (39). At both the N and C termini of the S protein are the signal and transmembrane peptides, respectively. These studies underscore the importance of the S protein in mediating disease severity and its therapeutic potential against SARS disease. We have characterized the key amino acid residues using an evolutionary RNA Qubevirus display strategy.

Qubevirus (Qβ) as a platform for display system: Qβ phages are small, positive-stranded RNA viruses that infect *Escherichia coli*; Qβ phages are found throughout the world in bacteria associated with sewage and animal feces (40). Each infectious phage Qβ is about 25 nm in diameter and belongs to the family of *Fiersviridae*. Qβ phages can express four genes within its 4220-nucleotide genome, that encodes (i) the subunit II (β) of replicase, (ii) a major coat protein (Cp), (iii) a maturation protein (A_2_ or MA_2_), and (iv) the minor coat or read-through protein (MCPA_1_ or A_1_) (41, 42, 43, 44, 45, 46). The life cycle of the Qβ phage starts with the adsorption of the phage on the bacteria's F+ pilus *via* the A_2_ protein, followed by injection of the RNA into the cytosol. The A_1_ protein shares the same initial codon with the Cp. This initial codon is produced during translation when the Cp stop codon UGA triplet is suppressed by a low level of ribosome misincorporation of tryptophan at the coat protein termination signal (47). Several functional peptides have recently been exposed on the Qubevirus platform through its A_1_ protein without affecting the phage viability, including a small biotin-tag (biot-tag) helping to sense any biotinylated entity (protein or nucleic acid) (48). In addition, the A_1_ display on Qubevirus and S protein exposed on the SARS-CoV occupied similar outer exposed positions. In addition, two epitopes buried in the S full length of SARS-CoV were mapped, and the chimera of all three-bearing fusion improved the binding to anti-S antibodies (27). The Qubevirus platform is then ideal to mimic the functionalities of the S protein domains.

In this report, the constructed recombinant phage vector plasmid harboring the small active receptor binding motif of the S protein of SARS-CoV was used to generate five different RBSM mutants; Y491F, D480N, N479V, N473E, and L472E, separately. Subsequently, all obtained plasmid mutants were expressed, and phages obtained were used for hACE2 target recognition analysis and anti-RBD antibodies against the S protein of SARS-CoV. Further, the Qβ phage displaying the spike RBSM blocked the infectivity of pseudo-SARS-CoV in a competitive assay, thereby effectively inhibiting viral entry by preventing the interaction between the spike protein and the host cell receptor. All five amino acid residues were thus essential and critical for the recognition and binding to the hACE2. Four of the five amino acid residues were essential and critical for recognition and binding to the anti-RBD antibodies of S protein. All chimera antigenic epitopes made of the three reported epitopes of SARS-CoV S protein and the signaling tag (Biot-tag and LPETG) were constructed and expressed. The Biot-tag was assessed to be the best transducer of signal for the chimeric epitope of S of SARS-CoV sensing anti-S antibodies with the best sequence being A_1_-Biot-tag-epitope3-epitope1-epitope2.

## Results

### Prediction of contact residues of the spike protein of SARS-CoV RBD shortest active motif and design of constructs on Qβ binding hACE2

**Figure 1:**
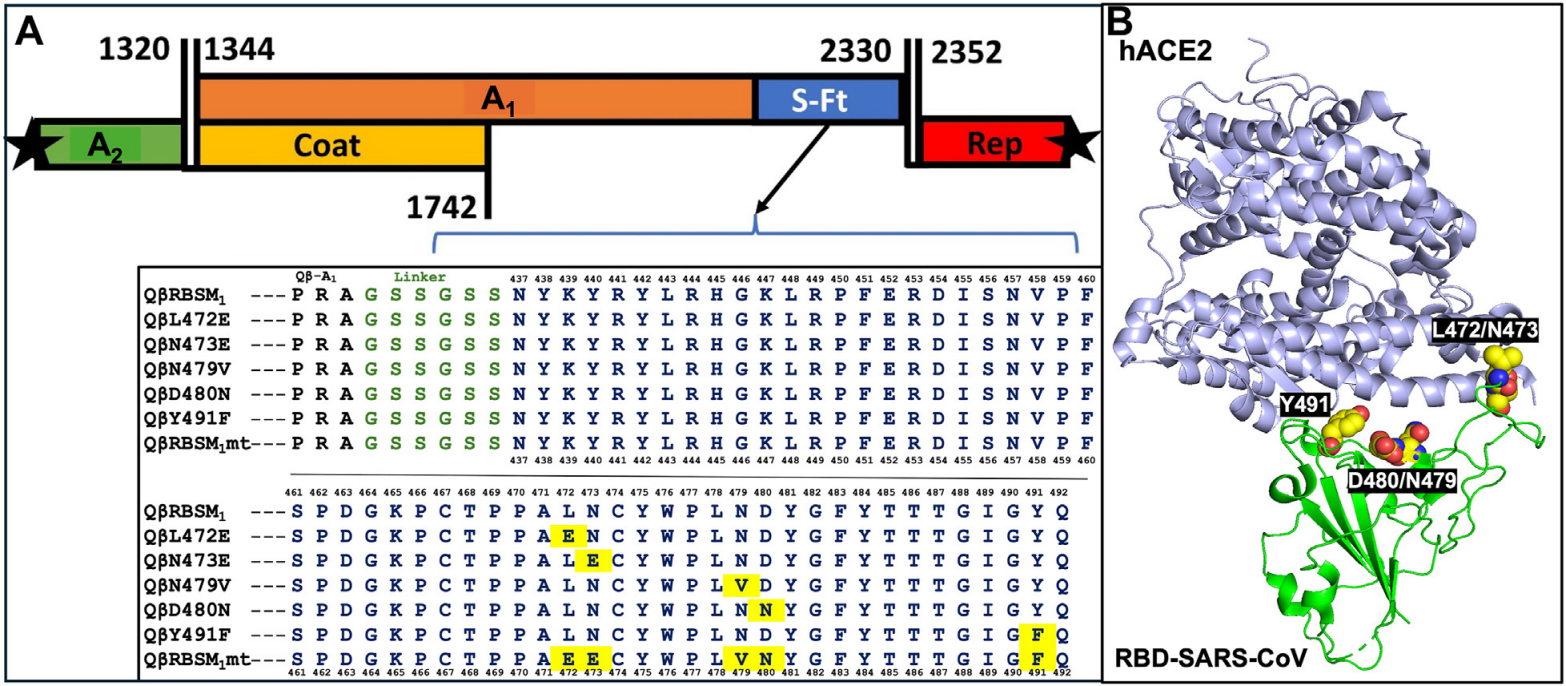
**Schematic representation of the RBSM mutant motif inserts into the A_1_ genome of Qβ, sequence alignment of the RBSM mutant motifs with the essential amino acid substitution mutations and the 3D structural simulation showing the binding contacts of the RBSM interacting with hACE2.***A*, *Up*: Qβ phage genome organization with the maturation protein (A_2_, *green*), the coat protein (*yellow*), the read-through protein (A_1_, *orange*) with the insertion cassette (S-Ft, *blue*) at the end for cloning, and the replicase protein (Rep, *red). Down*: aligned sequences of SARS-CoV RBSM (*blue*) showing the single essential amino acid substitutions (*yellow*) attached to a linker (GSS, *green*). QβRBSM1 is the recombinant Qβ phage harboring the SARS-CoV RBD motif. QβL472E, QβN473E, QβN479V, QβD480N, and QβY491F are recombinant QβRBSM1 phages with single amino acid substitutions at the indicated positions on the RBSM. QβRBSM1mt is the recombinant Qβ phage harboring the engineered RBD motif bearing all five mutations. Qβ is the WT Qβ phage used as control. *B*, interaction of the RBD (*green ribbon)* major essentials, L472, N473, N479, D480, and Y491 (*yellow, red,* and *blue spheres),* with hACE2 (PDB ID: 2AJF*light blue ribbon). h*ACE2, human angiotensin-converting enzyme 2; SARS-CoV, severe acute respiratory syndrome coronavirus; PDB, Protein Data Bank; RBD, receptor-binding domain; RBSM, receptor-binding small motif.

In our previous study, we mapped the shortest active hACE2 binding determinant that is situated between residues S_437–492,_ now called the RBSM on the spike (S) protein of SARS-CoV. In the crystal structure of the S RBD bound to hACE2 (Protein Data Bank (PDB) ID: 2AJF), we identified the following residues as potentially playing an important role in hydrophobic, hydrogen bonding, or charge interactions of RBSM with the hACE2 receptor: L472, N473, N479, D480, and Y491 (Fig. 1*B*). These residues fall within three distinct regions in the protein-protein interface between the RBD and hACE2. Four involve direct interactions with hACE2; these include L472 and N473 on one side of the interface, with N479 positioned in the middle region and Y491 on the other side. Although D480 does not directly interact with hACE2, it provides crucial electrostatic stabilization for N479. The following mutants were then designed: L472V, N473E, N479V, D480N, and Y491F, respectively, by single amino acid substitutions aimed at altering the properties of the targeted amino acids in the RBSM that interact with known residues of hACE2, in order to confirm the role of each amino acid.

In the crystal structure of the spike protein RBD bound to hACE2 (PDB ID: 2AJF), L472 engages in hydrophobic interactions with L79 and M82 of hACE2. The L472E variant would be expected to disrupt interactions with L79 and M82 of hACE2. The side chain -NH_2_ group of N473 hydrogen bonds to the -OH of Y83 and carbonyl (C=O) of Q24 in the receptor. The variant N473E would be charged but could potentially retain the hydrogen-bonding interactions of N473 (Fig. 3, *E* and *F*). Residue N479 is located within the spike protein RBD-receptor hACE2 interface close to H34 of hACE2. Adjacent to N479 is D480, which is a charged surface residue that does not directly interact with the receptor. The variant N479V places a hydrophobic residue within a fairly polar interface between RBD and the receptor, while the D480N variant eliminates a negative charge (Fig. 3, *C* and *D*). RBSM key residue Y491 forms a hydrogen bond through its -OH group with the carboxylate O of E37 residue within hACE2; E37 also hydrogen bonds to R393 of hACE2. Substitution of Y491 with F would preclude this hydrogen-bonding interaction and might significantly weaken the interaction (Fig. 3, *A* and *B*). In a bench-top assay to verify the effects of these mutations on the QβRBSM1, site-directed mutagenesis was used to generate these RBSM variants which were then displayed on the Qβ surface (Table 1). Previously, we demonstrated that the fusion prediction of A_1_ protein and the asexual stage antibody targeting protein of the merozoite surface antigen 3 (MSP3) could be modeled on the Qβ phage (49). We observed that the fusion model of the A_1_ protein with each single mutant separately was successfully generated and did not disturb the cloning cassette and the platform before the recombinant phage vector genetic construction.

**Figure 2:**
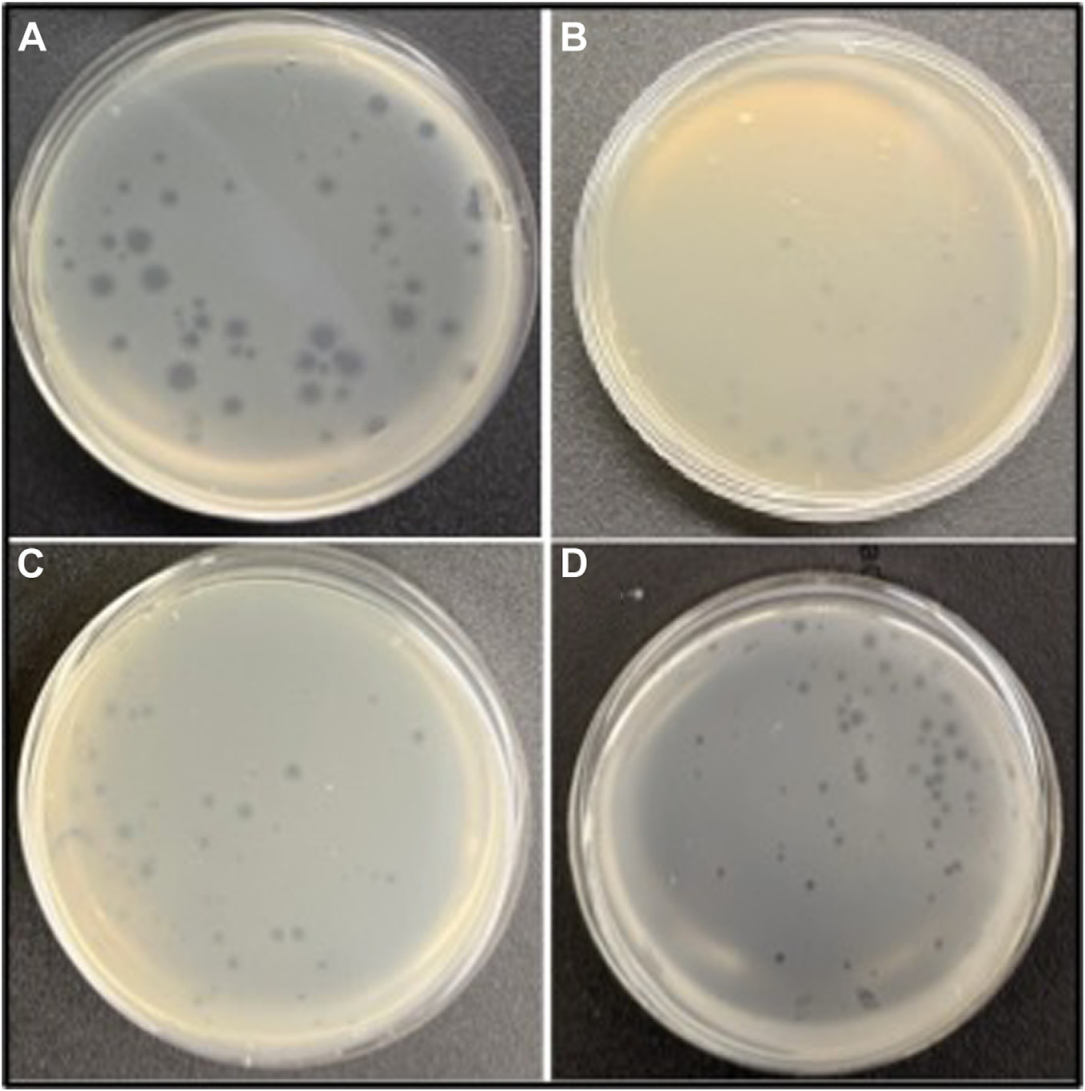
**Morphological analysis of the phenotype of the recombinant QβRBSM1 mutant phages**. The morphology of recombinant WT Qβ phage (*A*), the recombinant QβL472E phage (*B*), the recombinant QβY491F phage (*C*), and the recombinant QβRBSM1mt phage (*D*) on the lawn of the *Escherichia coli* Q13 host cell.

**Figure 3:**
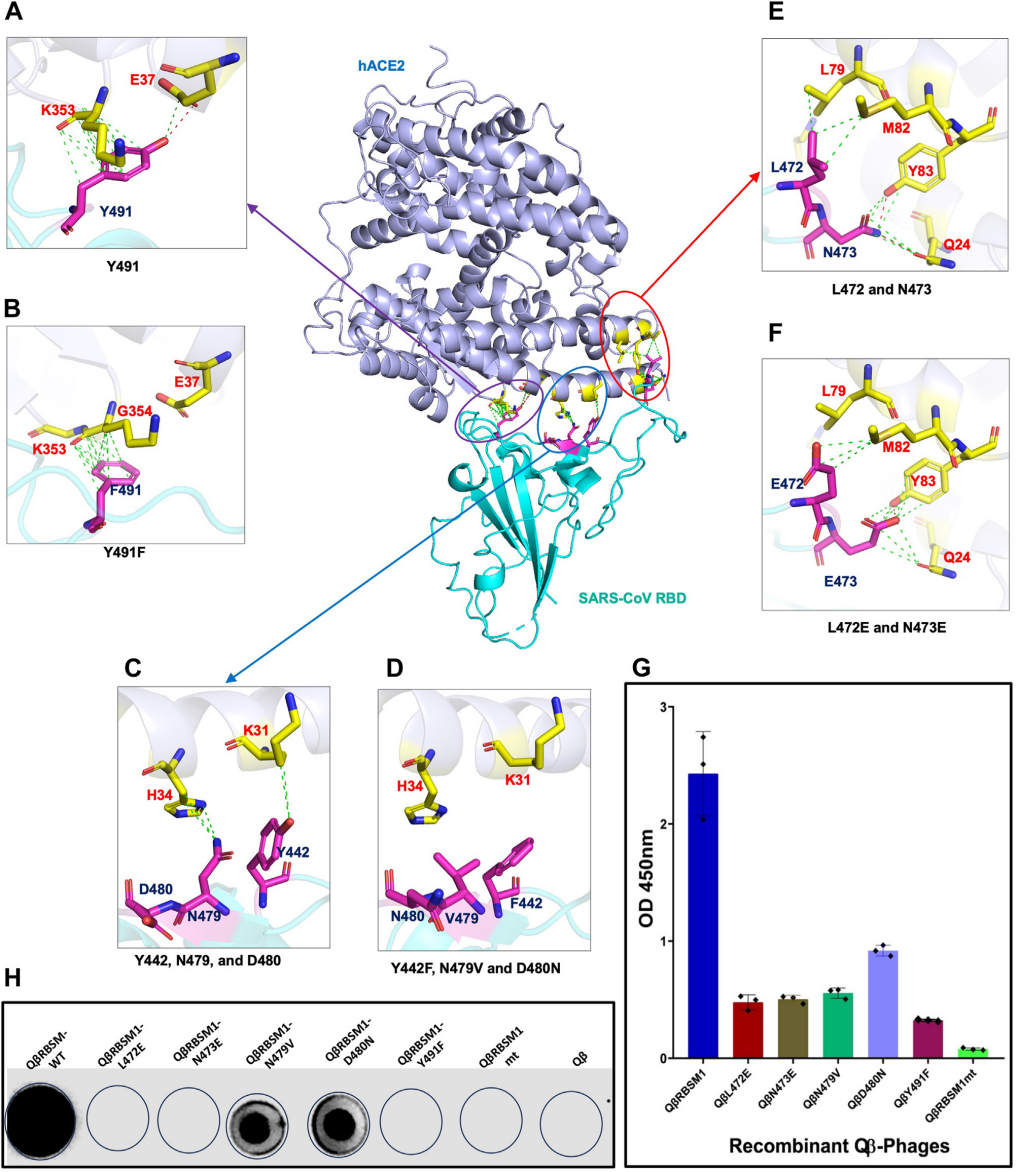
**Structural and functional characterization of Qβ-displayed SARS-CoV RBD motif mutants in hACE2 binding and antibody recognition.***A*–*F*, structural analysis of SARS-CoV RBD interactions with hACE2. The SARS-CoV RBD (*cyan*) is shown complexed with hACE2 (*light blue*), with key binding residues highlighted in *magenta* and corresponding hACE2 contact residues in *yellow*. *Red dotted lines* indicate key polar interactions, while *green dotted lines* represent additional molecular contacts. Close-up views illustrate the effects of specific mutations: *A*, WT Y491 interactions with hACE2, (*B*) altered interactions in the Y491F mutant, (*C*) WT Y442, N479, and D480 contacts with hACE2, (*D*) disrupted interactions in the Y442F, N479V, and D480N mutants, (*E*) WT L472 and N473 binding to hACE2, and (*F*) changes observed in the L472E and N473E mutants. *G*, ELISA analysis of recombinant Qβ phages displaying RBSM and mutant RBSM motifs. Binding of recombinant Qβ phages (10^2^ pfu/ml) to hACE2 was assessed for the WT RBSM1 and mutants (L472E, N473E, N479V, D480N, Y491F, and RBSM1mt). Data are presented as mean OD 450 nm ± S.D. from three independent experiments. *H*, dot blot analysis of recombinant Qβ phages for antibody recognition. WT Qβ and recombinant Qβ phages displaying RBD motif mutants (L472E, N473E, N479V, D480N, Y491F, and RBSM1mt) were analyzed for their recognition by SARS-CoV anti-RBD antibodies. The recombinant phages were tested at a concentration of 10^5^ pfu/ml. The ELISA test was performed in triplicate (n = 3), and the data are expressed as the mean ± standard deviation (mean ± SD). Statistical comparisons were carried out using one-way analysis of variance (ANOVA). hACE2, human angiotensin-converting enzyme 2; RBSM, receptor-binding small motif; SARS-CoV, severe acute respiratory syndrome coronavirus; RBD, receptor-binding domain.

**Table 1:** Comparison of titer and percentage binding activity to ACE2 relative to QβRBSM of the recombinant RBSM mutant Qβ phages

Recombinant phages	Phage titer (pfu/ml)	Binding percentage to hACE2 relative to QβRBSM1
QβRBSM1	10^7^	100
QβL472E	10^7^	19.7 ± 2.2
QβN473E	10^6^	20.7 ± 1.1
QβN479V	10^5^	22.9 ± 1.5
QβD480N	10^5^	37.8 ± 1.5
QβY491F	10^6^	13.3 ± 0.3
QβRBSM1mt	10^6^	3.2 ± 0.3

RBSM, receptor-binding small motif.

### Construction of phage vectors for the SARS-CoV spike gene receptor binding shortest active motif

We previously demonstrated that the SARS-CoV spike gene of RBD containing the shortest active motif (RBSM) of 55 amino acids could be obtained from the S fragment (F6) of 100 amino acids and built into the minor coat protein A_1_ platform for surface display upon recombinant Qβ phage (27). Using a similar approach in this study, the C terminus of the A_1_ protein platform was engineered to display all derived mutants of the RBSM of the spike protein of SARS-CoV (Fig. 1*B*). Since A_1_ is displayed on the surface of the phage particle, excessive modification by insertion of potential peptide motifs to be exposed might hamper the recombinant phage viability and production. For this purpose, it was necessary for us to examine and explore the tolerance of the Qβ genome for the insertion of new structures and longer RNA sequences. Before construction, we successfully obtained the predicted recombinant RNA secondary structure using the RNAfold (48) with codon optimization. We previously demonstrated stable insertion of a 150 to 300 bp DNA fragment (equivalent to 50–100 amino acids) into the Qβ complementary DNA (cDNA). The same procedure was thus sequentially used to generate recombinant phages bearing fragments of the S protein (27, 47, 48, 49). Our analysis confirmed that the RBSM and mutant genes fell within the specified size range and were not anticipated to increase the cDNA beyond the length of the recently fused A_1_ minor coat protein. During this process, restriction sequences of enzymes including Bpu10I, AflII, and NsiI were creatively built into both the insert (each RBSM mutant fragment) and the plasmid vector (pQβ8). The fusion PCR generated each RBSM mutant fragment with the appropriate size (∼800 bp) on the electrophoresis gel prior to plasmid vector construction (Fig. S1).

Likewise, plasmid vector expression cassettes were generated bearing an A_1_ gene modified in its C terminus to display RBSM mutant fragments of the S protein separately. As shown in Fig. S2, through sequential modification of the A_1_ genome, we have successfully inserted the DNA gene of each RBSM mutant, which was fused with its C terminus. The modified C terminus of the A_1_ genome was effectively fused with the 165 bp RBSM gene, and the tolerance of the Qβ genome for such a long-inserted DNA (300 bp) was established. Finally, a restriction site was built in between the A_1_ natural stop codons (TGA and TAA) and the NsiI cloning enzyme site prior to the clones/plasmids analysis. A total of seven fragment mutants of the RBSM peptide were effectively fused to A_1_ separately and built into the phage cDNA. In Figure 2*B*, a restriction enzyme gel analysis is shown for each fragment with the expected length. Contrary to previous reports (38), we found that the interregional section between A_1_ and the replicase genes was further extended rather than reduced. The various recombinant plasmids containing each set of RBSM mutation fragments were successfully sequenced, analyzed, and shown to contain the desired designed frame and relevant features necessary for the expression of recombinant phages.

### Production of recombinant Qβ phages displaying the RBSM mutants distinctly at various positions

Recombinant Qβ phages displaying the various RBSM mutants were produced using plasmid vectors separately constructed for each variant. These plasmids were transformed into *E. coli* DH5α or HB101, both of which are F^−^ bacteria lacking the pilus appendage required for reinfection. Also, the phage vectors are under the T7 promotor, which ensures exclusive usage of the recombinant DNA replication system with high-fidelity that leads to plasmid transcription, resulting in recombinant phage RNA genome production. The recombinant RNA genome obtained within the F^−^ bacteria was successfully used to avoid premature evolutionary events within the phage expression system and conserve the RBSM mutations created. Recombinant phages resulting from the expression of the recombinant plasmids with various mutations of the RBSM fragments, separately, were produced as plaques on lawns of their *E. coli* Q13 or K12 host (Fig. 2). Recombinant phage titers varying between 10^5^ and 10^7^ pfu/ml were obtained for various fragment mutants, respectively (Table 2). The fragment mutants QβRBSM1-N479V and QβRBSM1-D480N were poorly expressed and generated the lowest phage titer. The genome of each mutant variant was analyzed by reverse transcription-polymerase chain reaction (RT-PCR), agarose gel electrophoresis, and sequencing reactions. The results showed that each variant containing the expected DNA fragment size (Fig. S3) when compared to the Qβ phages fused with a Vg2 gene, and the control phage with a deleted A_1_. A fragment size of 1800 bp was formed consisting of the A_1_, RBSM mutant fragment, interregional section upstream of the replicase, and partial replicase genes, respectively. To confirm the presence of the corresponding RBSM mutant fragment gene within the plaques obtained, each phage variant's cDNA was sequenced. The results show that recombinant phages from each plaque corresponded specifically to their respective gene (genotype) fused in frame with the A_1_.

**Table 2:** List of oligonucleotides names and sequences with bold are the phages A_1_ gene portions

Mutant and restriction enzyme	Reverse primer sequence for mutant construction
Y491F *Nde1*	gaactgctattggccgctgaacgattatggcttttataccaccggcattggctTtcagTGATAAgcATATGCctaaggatgaaatgcATG
D480N *Not1*	gaactgctattggccgctgaacAattatggcttttataccaccggcattggctatcagTGATAAgcggccGCctaaggatgaaatgcATG
N479V *Nhe1*	gcgctgaactgctattggccgctgGTcgattatggcttttataccaccggcattggctatcagTGATAAgcggccGCctaaggatgaaatgcATG
N473E *ECORV*	gccggcgctgGaAtgctattggccgctgaacgattatggcttttataccaccggcattggctatcagTGATAAgATATcGCctaaggatgaaatgcATG
L472E *PST1*	gccggcgGAgaactgctattggccgctgaacgattatggcttttataccaccggcattggctatcagTGATAACTGCAGctaaggatgaaatgcATG

## Contribution of the predicted amino acid residues of RBSM of SARS-CoV to the recognition of the hACE2 receptor

We previously demonstrated an ELISA strategy with recombinant hACE2 (rhACE2) to measure the affinity of a synthetic S fragment motif derived from F6. In the study, rhACE2, the natural receptor of SARS-CoV, was immobilized as the target prior to the selective analysis *via* quantitative ELISA. Through this ELISA process, the level of affinity of each set of recombinant phages harboring RBSM of the SARS-CoV S toward rhACE2 was quantified with the previously reported phages; RBSM served as the control and standard. The same phage titer, RNA, and protein concentration were used for each RBSM mutant. Firstly, the affinity of the recombinant phages presenting Y491F and L472E within the RBSM for rhACE2 was drastically reduced to 13% and 17%, respectively, compared to the RBSM control. Secondly, phages displaying N473E and N479V showed a 20% reduction in affinity relative to the RBSM standard (Table 1). Thirdly, the D480N mutation resulted in a 37% reduction in affinity. Lastly, phages harboring the RBSM with all mutations together (RBSM1mt) exhibited an almost complete loss of binding, with only 3% affinity remaining compared to the RBSM standard (Fig. 3*G*).

Overall, the affinity of single mutations of the SARS-CoV RBSM for the hACE2 ranges between 13 and 35%, while the combination of all five mutations decreased to 3%, highlighting their synergetic effect on the initiation of the infection (Fig. 3, *A*–*G*). These studies demonstrated that the Qβ phage displaying RBSM (phenotype) can be utilized to effectively mimic and determine the five important amino acid residues of S of SARS-CoV initiating the infection.

### Analysis of recombinant phages harboring the S protein RBSM mutants against SARS-CoV anti-RBD antibody

**Figure 4:**
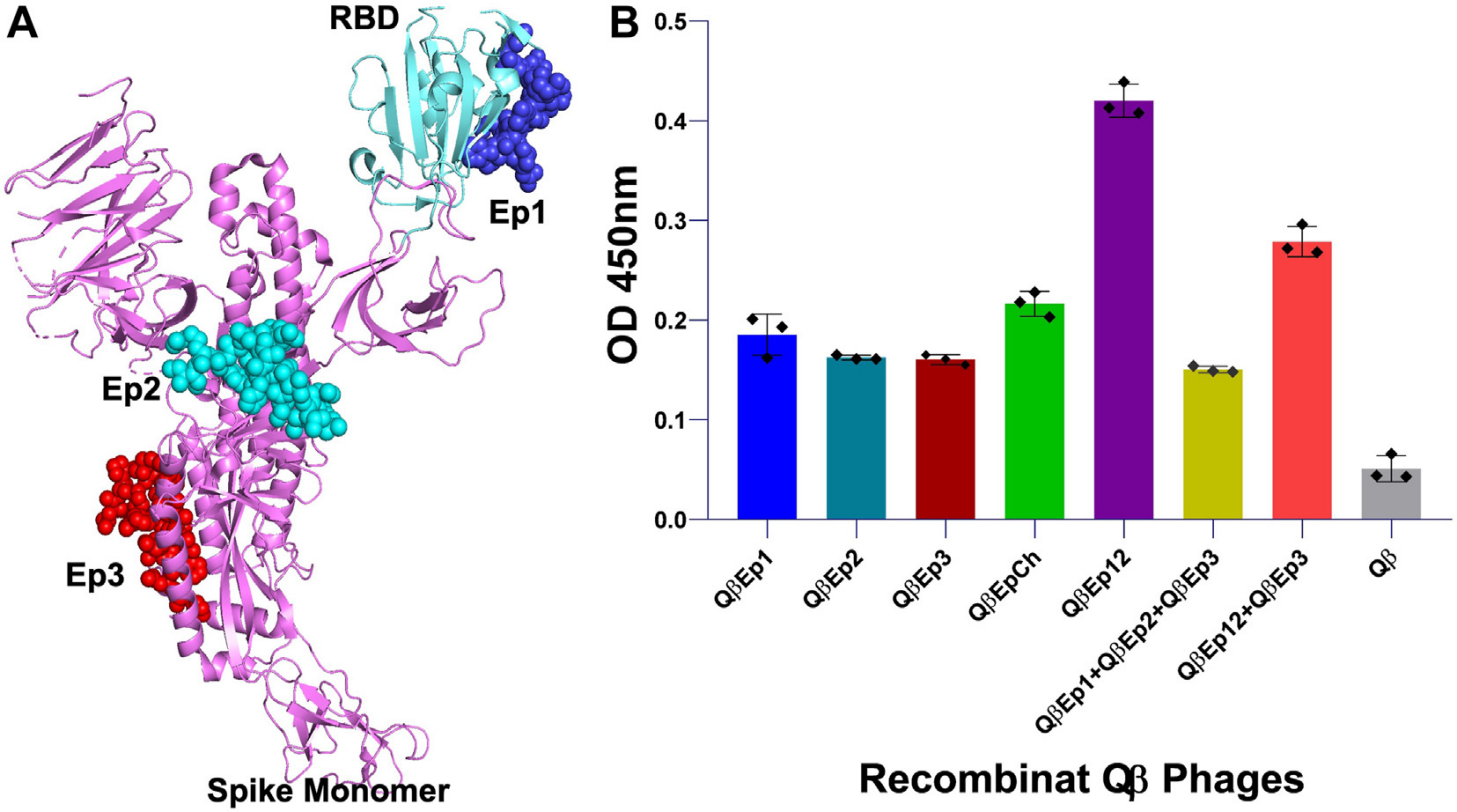
**SARS-CoV S protein mapped with epitopes showing binding analysis of the exposed epitopes against anti-S antibodies.***A*, the image on the *left* shows the SARS-CoV spike protein monomer (*pink ribbon*, PDB ID: 8H15) and RBD (*light cyan ribbon*) with epitopes Ep1 (*blue*), Ep2 (*cyan*), and Ep3 (*red*) in *spheres*. *B*, ELISA analysis of recombinant Qβ phages displaying the S-protein-selected epitopes (Ep). Ep1 (F6: S_425–460_); Ep2 (F7/F8: S_601–620_); Ep3 (F10: S_781–800_); EpCh (chimeric of Ep1, Ep2, and Ep3); and Ep 12 (chimeric of Ep 1 and Ep 2). The control is the WT Qβ phage. The titer of recombinant phages was 10^3^ pfu/ml. The ELISA test was performed in triplicate (n = 3), and the data are expressed as the mean ± standard deviation (mean ± SD). Statistical comparisons were carried out using one-way analysis of variance (ANOVA). SARS-CoV, severe acute respiratory syndrome coronavirus; PDB, Protein Data Bank; RBD, receptor-binding domain.

To further determine the contribution of each predicted amino acid residue of the SARS-CoV during infection initiation, recombinant phages presenting various S RBSM fragment mutants, respectively, were incubated with anti-RBD antibodies (Fig. 3*H*) in a dot blotting assay. Using the QβRBSM1 as a control and standard, QβRBSM1-N479V and QβRBSM1-D480N reacted poorly with the anti-RBD antibody, while the recombinant phages harboring other mutations in occurrence, L472V, N473E, and Y491F, did not react with the anti-RBD antibody. This result demonstrates that the residues L472, N473, and Y491 are essential for the anti-RBD specific epitope. In an earlier experiment where these residues of the RBSM were mutated and tested for recognition and binding to the hACE2, a similar pattern was observed and related to all five mutants together. The residues L472, N473, and Y491 seem essential in hACE2, and anti-RBD antibody recognition and binding. Moreover, the recombinant phages displaying RBSM mutant fragments showed no effect on the viability, structure, morphology, and stability when compared to the WT phage platform that enhanced the functionality of the peptide displayed (Fig. 4).

### Contribution of each S protein epitope to the anti-S antibody neutralization and positioning on the phage Qβ platform

**Figure 5:**
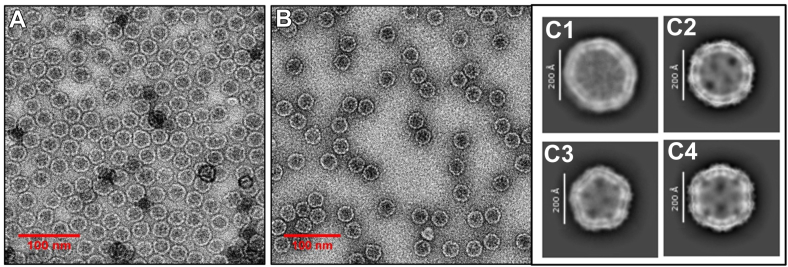
**Electron microscope images of phages harboring the SARS-CoV spike protein RBSM.***PanelA*: negative stain transmission electron microscope images after CsCl gradient purification and dialysis of Qβ WT (more confluent 108 pfu/ml); *PanelB*: QβRBSM1 (less confluent 106 pfu/ml). *PanelC*: transmission electron microscopy initiating the cryo-EM of C1: Qβ WT phage; C2: QβRBSM1; C3: QβRBSM1mt; C4: QβRBSM1mt2 (Y491F and N473E). QβRBSM1 is the recombinant Qβ phage harboring the SARS-CoV RBD motif. QβRBSM1mt is the recombinant Qβ phage harboring the engineered RBD motif (RBSM) bearing all five mutations (L472E, N473E, N479V, D480N, and Y491F) of each single amino acid substitution at the indicated position on the RBD motif all together. As indicated, mt2 in QβRBSM1mt2 is with two amino acids mutation: Y491F and N473E. The concentration of recombinant phages was 103 pfu/ml, respectively. RBSM, receptor-binding small motif; RBD, receptor-binding domain.

We have recently mapped three major epitope regions on the S protein of SARS-CoV to be situated at residues AA 441 to 460 for epitope 1 (Ep1), 601 to 620 for epitope 2 (Ep2), and 781 to 800 for epitope 3 (Ep3), respectively, that were all confirmed by monoclonal antibodies from patient-derived samples from National Institutes of Health (50). We reconstructed a set of recombinant phages, each harboring epitopes individually or in combination, for quantitative ELISA analysis under the same titer, RNA, and proteins. Ep1 was the only epitope localized on the interface of S protein interacting with hACE2 (Fig. 5*A*), that played a dual role in initiation of infection through binding to it and involvement in antibody production. As previously described, Ep1 showed strong binding activity to S-specific antibodies, followed by Ep2 and Ep3 (Fig. 5*B*). In addition, chimera constructs with different epitope positioning showed different affinity reactivities. The previously reported chimeric epitope (EpCh) enhanced antibody recognition, whereas a mixture of recombinant phages displaying individual epitopes separately (QβEp1 + QβEp2 + QβEp3) under the same volume, titer, RNA, and protein concentrations did not show any significant change in antibody recognition. Notably, the chimera of Ep1 and Ep2 linked with their last and first 25 natural amino acids (Ep12) showed the best and substantially improved recognition and binding to the cognate anti-S antibodies. A mixture of the recombinant QβEp12 with QβEp3, tested under the same volume, titer, RNA, and protein concentration conditions, in a quantitative ELISA exhibited improved affinity to anti-S antibodies compared to QβEpCh, though slightly lower than QβEp12 alone. Altogether, the results demonstrated that epitope foldability is preserved on the Qβ platform, and that accessibility is essential for chimeras with short linker peptides. The differences in recognition and binding activity of recombinant phages harboring the chimeric epitopes to anti-S antibodies indicates that these recombinant phages successfully display properly folded functional peptide sequences (phenotype) defined by the inserted gene into the phage genome (genotype). Hence, the direct correlation between the phenotype and genotype validates the Qubevirus platform for precise protein fine functionality.

### Construction and analysis of recombinant phages bearing the chimeric epitope positioning with the Biot-tag or LPETG tags

**Figure 6:**
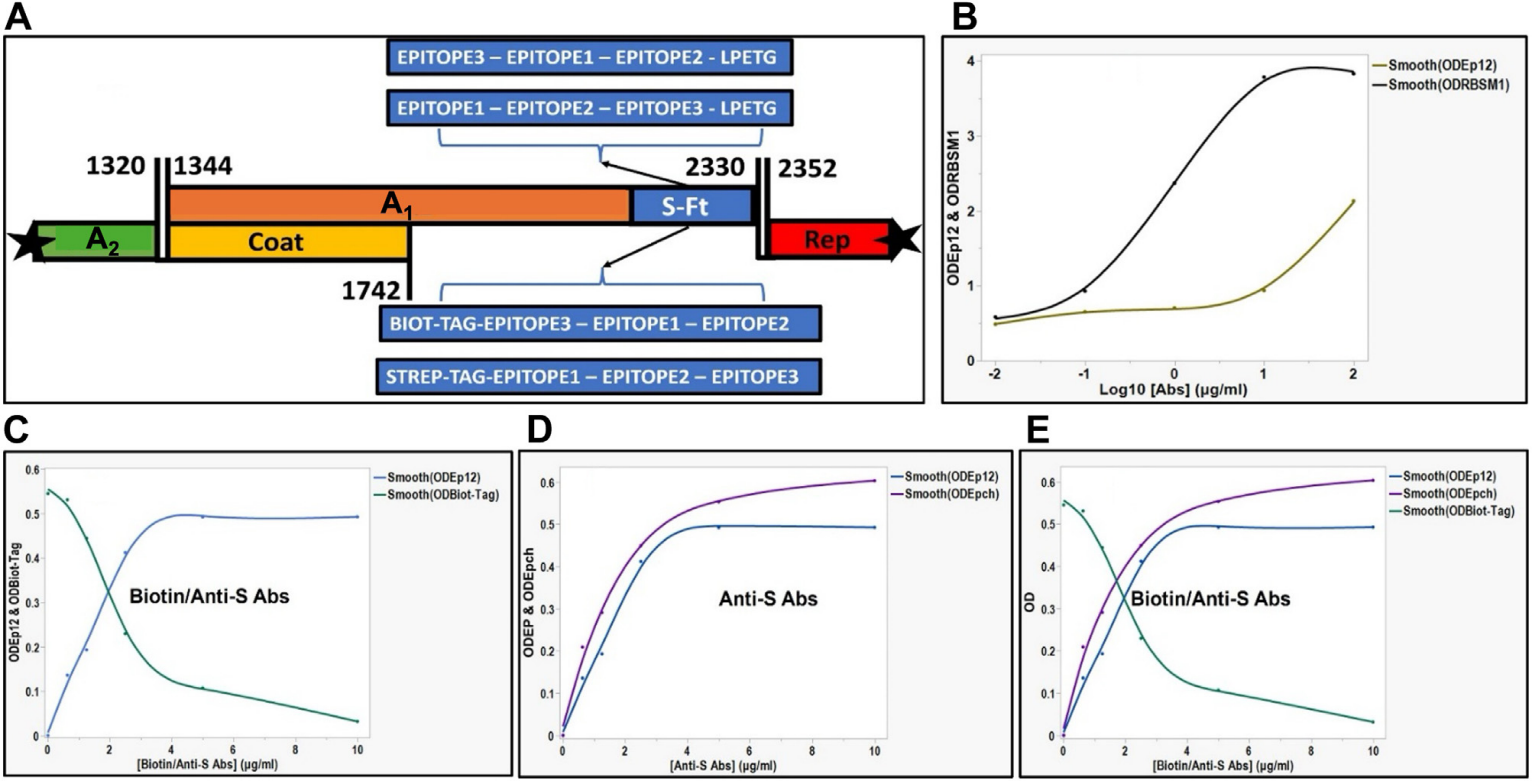
**Schematic representation of the recombinant Qβ phage cDNA organization with the positioning and reactivities of chimera and various transducer peptides.***A*, Qβ phage genome organization with the maturation protein (A_2_, *green*), the coat protein (*yellow*), the read-through protein (A_1_, *orange*) with the insertion cassette for S fragments (S-Ft, *blue*) at the C terminus for cloning, and the replicase protein (Rep, *red*). The chimera of epitopes with transducer organization are indicated in order from *left* to *right* within the positioning to form the different biosensors; tags LPETG and biotin Tag (Biot-Tag) separately at the N and C termini of the chimera epitopes, respectively. *B*, ELISA with recombinant phages harboring the RBSM1 and the chimeric epitope1 and epitope2 (Ep12; epitope1-epitope2 with two-thirds of natural amino acid between as linker) against SARS-CoV anti-S antibodies and biotin-HRP, respectively; graphical representation of the optical density (OD) of the horseradish peroxidase (HRP) product of anti-S antibodies against the increased concentrations of the anti-S antibodies on a plate coated with 10^3^ pfu/ml of the recombinant phages (*black line*: RBSM1 and *light green line*: Ep12). *C*, biotin-HRP: the plot analysis of OD versus increase of concentration of biotin conjugated to HRP (plate coated with 10^3^ pfu/ml Qβ-Strep). *D,* the Ep12 chimeric anti-S antibodies plot of the absorbance of the HRP product of anti-S-antibodies against increased concentration of the anti-S antibodies (plate coated with 10^3^ pfu/ml Qβ-Ep12). *E*, biotin-HRP: the plot analysis of OD *versus* increase of concentration of biotin conjugated to HRP (plate coated with 10^3^ pfu/ml Qβ-Biot). cDNA, complementary DNA; RBSM, receptor-binding small motif.

In an effort to determine the optimal positioning of EP1, EP2, and EP3 on the Qβ platform, chimera constructs incorporating all three epitopes (EPCh) were systematically designed. First, the peptide LPETG was fused sequentially to each chimeric construct, followed by random positioning of epitopes, joined by linkers, and subsequently analyzed (Fig. 6*A*). The peptide LPETG motif is specifically recognized by the enzyme sortase A (SrtA), enabling ligation to a fluorescently measurable nanobody at the C terminus of the chimeric epitope. This LPETG-chimeric-nanobody complex allowed for the determination of the optimal positioning of EP1, EP2, and EP3 based on maximal antibody recognition and binding and the minimum fluorescence signal. In addition, a Biot-tag peptide (15-mer recognizing and binding biotin), was used for the same purpose. The highest signal with the anti-S antibody was observed when EP3 was positioned closest to the A_1_ protein C-terminus, followed by EP1 and EP2. This trend was further validated by quantitative ELISA (Fig. 6, Panel *C* and *D*), where the chimeric construct with EP1 and EP2 more exposed showed the highest absorbance, compared to any other positioning of individual constructs (EP1, EP2, and EP3) displayed on the phage surface.

### Inhibitory and cytotoxic effects of recombinant phages displaying SARS-CoV receptor binding small motif (QβRBSM1)

**Figure 7:**
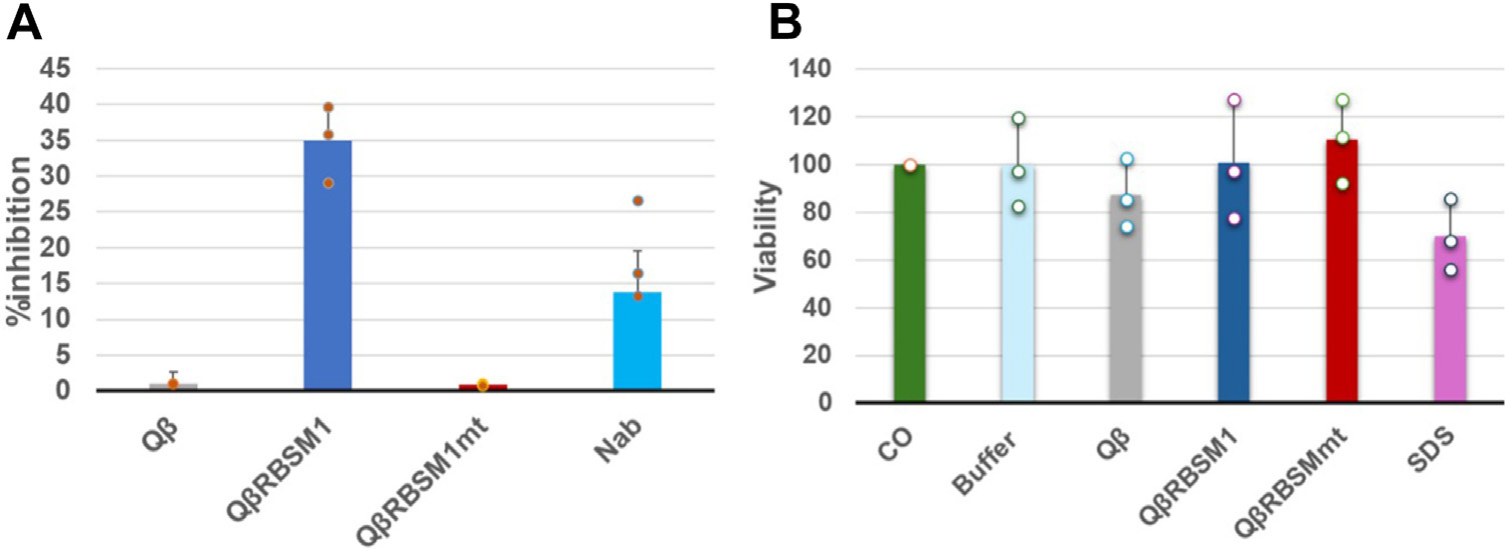
**Inhibitory effects and cytotoxicity of recombinant Qβ phages.***A*, inhibition of SARS-CoV pseudovirus by recombinant Qβ phages and anti-SARS-CoV receptor-binding domain (RBD) antibody (Nab). Qβ is WT phage; QβRBSM1 and QβRBSM1mt are recombinant phages. Nab is antibody against the SARS-CoV receptor-binding domain. *B*, cytotoxicity of Qβ phages on HEK293T cells. CO represents cells cultured alone. CO and phage buffer were used as negative controls, while SDS served as positive control. Qβ is WT phage; QβRBSM1 and QβRBSM1mt are recombinant phages. The test was performed in triplicate (n = 3), and the data are expressed as the mean ± standard deviation (mean ± SD). Statistical comparisons were performed using one-way analysis of variance (ANOVA).

To further validate the *in vitro* binding affinity of the RBSM displayed on Qβ phage platform to the host receptor ACE2, a competitive assay was performed against SARS-CoV pseudovirus. Interestingly, the recombinant phages CsCl purified, dialyzed, and bearing the native receptor binding motif (QβRBSM1) were shown to significantly block the viral infectivity with a percentage of inhibition of 34.90% when tested at a titer of 10^7^ pfu/ml (Fig. 7*A*). Meanwhile, the phages exposing the receptor binding motif with all the mutated key amino acids (QβRBSM1mt) did not exhibit inhibition like the WT Qβ phages in comparison to the QβRBSM1.

On the other hand, the recombinant phages did not affect cell viability compared to the control (cells only) although QβRBSM1mt slightly stimulated cell growth. WT Qβ phages slightly reduced cell viability by approximately 15%, though the effect was not significant when compared to the positive control (SDS) that decreased the number of viable cells by about 33% (Fig. 7*B*). These results showed that recombinant phages at a titer up to 10^7^ pfu/ml can efficiently inhibit SARS-CoV infection without compromising host cell viability.

## Discussion

The SARS disease outbreak of 2002 caused by SARS-CoV raised significant scientific molecular and immunological concerns. Knowing the pivotal role of the S protein in mediating viral cell entry and having mapped its epitopes and ACE2-binding fragment, we aimed to determine the immunological significance of each epitope and assess the contribution of key residues within the RBSM to viral tropism. Other than the tropism, the S protein with the three mapped epitopes (S_441–460_, S_601–620_, and S_781–800_) is immunogenic and a target for neutralizing antibodies for therapeutic purposes. Furthermore, chimeric constructs of these epitopes, fused with a transducer tag on the Qβ phage platform, offer potential as tools for anti-S antibody titration. Using the Qubevirus display system, our data directly demonstrates the functional relevance of RBSM mutants with recombinant hACE2, anti-S, and anti-RBD antibodies. The recombinant phage displaying the RBSM effectively competed with the SARS-CoV S protein for hACE2 binding, highlighting its potential as a therapeutic for SARS disease. The sequence of epitopes in the chimera can be used as a point-of-care diagnostic biosensor toolkit. In this study, we predicted, and validated five essential residues (L472, N473, N479, D480, and Y491) involved in hACE2 recognition and binding. Their respective variants (L472E, N473E, N479V, D480N, and Y491F) abolished the RBSM's ability to bind hACE2 and neutralize anti-RBD antibodies. In addition, Qβ-displayed RBSM significantly inhibited SARS-CoV pseudovirus entry in a competitive assay, whereas the mutant version showed minimal inhibition. These findings highlight the crucial role of these five residues in initiating infection and defining immunogenic determinants. We evaluated the immunogenicity of each epitope, and the combinations thereof, particularly Ep1 and Ep2 (S_441–460_ and S_601–620_) to understand their structural and functional rules. Collectively, these findings offer valuable insights for SARS-CoV subunit vaccine development, antiviral peptide design, entry inhibition, and antibody titration toolkits. In addition, they help define the functionality of the S protein in infection and evolutionary studies.

Among the key residues identified on the RBSM (S_437–492_), five were identified as essential for surface contact and binding with hACE2. In a comparative structural analysis of SARS-CoV and SARS-CoV2 spike proteins (9), three contact regions (CR1 to CR3) between RBD and hACE2 were delineated: CR1 (L472/N473 in SARS-CoV and F486/N487 in SARS-CoV-2), CR2 (N479/D480 in SARS-CoV and Q493/S494 in SARS-CoV-2), and CR3 (Y491 in SARS-CoV in and Y505 in SARS-CoV-2). In SARS-CoV, L472 interacts with L79/M82, and N473 forms hydrogen bonds with Q24/Y83 of hACE2. N479, while not forming direct hydrogen bonds, interacts with H34, and D480 does not form any direct contact with hACE2, while Y491 forms a hydrogen bond with E37. Despite sequence differences, SARS-CoV-2 residues mediate similar interactions. In SARS-CoV-2, F486 is sandwiched between M82/Y84, N487 bonds with Q24/Y83, Q493 interacts with E35, and S494 does not contact hACE2 directly but bonds internally to Y436. Y505 mirrors Y491 in contacting E37 (9, 30).

Mutation of all five residues abolished the capability of SARS-CoV RBSM to mediate tropism and recognize anti-RDB antibodies. D480, with no direct hACE2 contact, had the least impact (40% residual binding after D480N mutation). Residues L472 and Y491, located within the two primary contact regions, contributed significantly to hACE2 binding, followed by residues N473 and N479. Site-directed mutagenesis of a single L472E or Y491F mutation reduced binding to 13 to 17%, and their combination nearly matched the loss-of-function observed in the full quintuple mutant (RBSM1mt), underscoring their critical role in receptor recognition and binding. Our findings do not exclude the possibility that simultaneous deletion of both residues may induce conformational changes within the RBSM as previously reported (51, 52). Moreover, in a competition assay using SARS-CoV pseudovirus, the recombinant phage QβRBSM1 effectively blocked SARS-CoV pseudovirus entry, whereas the recombinant phage mutant QβRBSM1mt exhibited minimal inhibition. This highlights the critical role of these residues in host receptor recognition and binding. The mutation of these residues significantly impairs viral interaction with hACE2 receptor, reinforcing their importance in SARS-CoV infectivity. As a result, the potential relevance of phage-displayed RBSM in mediating viral inhibition is well highlighted, suggesting a potential application in antiviral interventions. Importantly, cytotoxicity assays confirmed that both WT and recombinant phages exhibited minimal cytotoxic effect on host cells, maintaining an acceptable safety profile. With historical and regulatory support for phage therapeutics (53, 54), phage-displayed RBSM (particularly constructs like QβRBSM1) emerge as promising antiviral candidates, capable of competitively inhibiting SARS-CoV binding to hACE2.

Previous computational studies have equally identified multiple contact residues at the SARS-CoV RBD-ACE2 interface, highlighting key interactions that contribute to receptor binding (30). Beyond the five key amino acids examined in this study, substitutions at other residues within the binding surface of the SARS-CoV RBD, namely: Y440, Y442, L443, Y475, and Y484 do not appear to substantially reduce hACE2 binding. Notably, Y440 and Y475 are conserved between SARS-CoV and SARS-CoV-2, and mediate hydrogen bond interactions with hACE2. These findings indicate that substitutions at these positions, as long as they preserve these crucial interactions, are unlikely to significantly disrupt RBSM-hACE2 binding (9, 11, 30).

In our previous work, we mapped the antigenicity of the S proteins of SARS-CoV by identifying specific epitopes that play key roles in immune recognition and binding (27). Using a library of sequential S fragment deletions displayed on the Qubevirus platform, we localized three major linear epitopes, namely: Ep1, Ep2, and Ep3. Each retained its conformational integrity when fused to the A_1_ protein on the Qβ platform and displayed distinct binding reactivity toward the S protein-specific antibodies resulting from the absorbance (*A*) measurement from enzymatic assays. These differences in reactivity likely reflect structural complementarity between each epitope and its cognate antibody. Notably, Ep1 (S_441-460_), is located within the RBSM, a region we previously showed to contain an immunodominant neutralizing epitope with low affinity for the cognate anti-S antibody. This affinity was enriched only when the Ep1 region was well-exposed on the Qubevirus platform (27). Our data suggest that the S protein likely undergoes internal molecular rearrangements before and after tropism to expose these epitopes that are not important during infection initiation and hACE2 recognition and binding. Prior studies demonstrate the biological impact of amino acid mutations by deletion or substitution within the RBD across coronaviruses (4). Furthermore, a chimeric S protein epitope (EPCh), incorporating three optimized epitopes, was engineered, expressed, and evaluated for antibody reactivity as previously described (27). In this study, we determined the contribution of each epitope and its positioning within the chimera. The chimeric epitope showed better affinity for the cognate anti-S antibody in comparison to each individual epitope. The affinity was improved with the Ep3 directly fused to the A_1_ protein, followed by the Ep1 and Ep2, highlighting the importance of positioning for optimal conformational structure and epitope accessibility on the Qβ platform. Linker analysis revealed that long-arm linkers enhanced antibody binding, suggesting improved epitope exposure. A refined construct, Ep12, was created by retaining two-thirds of the residues of the S protein fragment between Ep1 and Ep2. Ep12 showed superior antibody affinity and reactivity, identifying it as a major immunodominant region of the SARS-CoV S protein. Its natural linkage likely supports a conformation conducive to neutralizing antibody recognition. We speculate that this major immunodominant neutralization domain has both the appropriate accessibility and conformation. When displayed on Qβ, Ep12 mimics the native immunogen and could serve as a subunit vaccine candidate, alone or in combination with Ep3. Given the A1 protein localizes at the 12 vertices of the Qβ icosahedron (55), each phage particle could present 12 copies of the chimera, amplifying immunogenicity.

We further explored functional accessibility by fusing a transducer peptide, the biotin-tag (Biot-tag), to the chimera. Our data showed that the transducer peptide, Biot-tag, recognizes and binds to the cognate biotin conjugated to horseradish peroxidase (HRP), regardless of its position. Competition assays revealed that increasing concentrations of anti-S antibodies progressively reduced Biot-tag accessibility, and *vice versa*. At 3 mg/ml, biotin blocked antibody access, while 4 mg/ml of anti-S antibody saturated 10^3^ pfu/ml of recombinant phages exposing the chimeric epitope of SARS-CoV S protein. These results suggest steric hindrance or conformational interference when multiple cognate molecules target adjacent regions. The same phenomenon was observed with sortase A-mediated ligation of an LPTEG peptide tag, where the C terminus of the chimera was found to be the best positioning for the LPETG tag and enzyme accessibility. The competition between proteins for a site on the Qβ platform was recently observed for the foot-and-mouth disease virus (FMDV) epitope using the cognate SD6 monoclonal antibody and the Strep II tag and streptavidin (50). Thus, spatial proximity of the chimeric epitope and the transducer, binding to one cognate protein abrogates access by its competitor, and saturation thresholds must be determined empirically. The combination of SARS-CoV chimeric epitope on the phage surface fused with transducer peptide can be a toolkit to titer specific anti-S antibodies in any plasma sample.

In conclusion, we have identified through recombinant Qβ phage mimicry, the functional characterization of the S protein-specific small domain (RBSM) in binding hACE2 and the essential residues, namely, L472 and Y491, among the five projected from the structure. These residues are crucial and in addition to those previously predicted and reported. This Qβ phage-displayed RBSM has the potential to block SARS-CoV infection in a competition assay with the SARS-CoV pseudovirus and can be a therapeutic candidate for SARS treatment. In addition, three epitopes, recently confirmed by antibodies from SARS patients, were engineered and positioned on the Qβ platform together with a transducer to serve as a biosensor toolkit for specific anti-S antibody titration. Recombinant Qβ phages displaying the chimeric epitope of Ep1 and Ep2 (Ep12), linked by two-thirds of the S protein's natural amino acid residues between positions 461 and 600 were also described. These recombinant phages bearing the chimeric epitope Ep12, selectively bind to the cognate anti-S antibodies. Thus, our results provide useful biochemical insights into an in-depth understanding of the SARS-CoV S protein and its potential exploitation for the development of point-of-care detection toolkits, as well as prophylaxis and therapeutics against SARS-CoV infection. Similarly, we are extending this investigation to the SARS-CoV-2 S protein, considering the related sequence and its role in all vaccines currently in use.

## Experimental procedures

### Material

#### Plasmids, bacteria, antibodies, and rhACE2

The Qubevirus plasmids (pQβ7, pQβ8, and pQβAd2) from the Waffo lab at Indiana University School of Medicine were used for the construction of the recombinant derivatives bearing the RBSM and its corresponding mutants as inserts. For subcloning, phage expression, and phage amplification, *E. coli* strains, HB101, DH5α, and Q13, respectively, were used. The antibodies were purchased from ABClonal (Cat # A20135, AS014, and A21181). The rhACE2 was purchased from Sino Biological (Cat # 10108-H08H). The SARS-CoV pseudovirus neutralization kit was purchased from Virongy Biosciences.

### Methods

#### Contact residues prediction between S and hACE2 proteins

Spike protein RBD residues involved in direct interactions with hACE2 were identified through inspection of the protein-protein interface in the crystal structure of the SARS-CoV RBD-hACE2 complex (PDB ID: 2AJF). Comparative structural analysis for SARS-CoV2 RBD bound to hACE2 was done by superimposing its structure (PDB ID: 6MOJ) with that of SARS-CoV (PDB ID: 2AJF).

#### Construction of recombinant plasmids with various RBD mutants

Fusion PCR was used to generate each RBSM mutant insert. pQβRBSM1, initially constructed by our group, was used as the template. It contained a sequence of the smallest active spike (S) protein binding motif (RBSM) of the SARS-CoV, the linker peptide, and the C terminus of the A_1_ protein of Qβ, in frame with each other, respectively. The forward primer contained the AflII restriction site and a portion of the A_1_ sequence of Qβ, whereas the reverse primer for each mutant (L472E, N473E, N479V, D480N, and Y491F) contained the part of the RBSM fragment containing each of the mutations, the two natural stop codons of A_1_ (TGATAA), a restriction site (NotI, PstI, EcoRV, NheI, NotI, and NdeI, respectively), and the Nsi*I* restriction site. For each insert bearing the intended mutation, the PCR product (∼400 bp) was extracted using agarose gel electrophoresis, digested with AflII and NsiI, and purified. The RBSMmt bearing all the five mutations was synthesized into the pUCS plasmid containing the AflII restriction site, the entire RBSM gene with all the five mutations, the two natural stop codons of A_1_, the NotI restriction site, and the NsiI restriction site. The insert was obtained by restriction digestion of the pUCS plasmid using AflII and Nsi*I* and purified. For cloning, the pQβ7 vector was restricted with AflII and NsiI, dephosphorylated with Quick CIP and purified. The purified vector and each insert were separately ligated overnight at 16 °C (48). Each ligated product (20 μl) was used to transform *E. coli* HB101-competent cells. The transformed cells were plated in 2YT agar supplemented with 100 mg/g of ampicillin. For each of the transformations, 10 clones were picked and used to prepare DNA for screening. The DNA obtained was screened using the restriction enzymes added before the NsiI cloning site (NotI, PstI, EcoRV, NheI, NotI, NdeI, and Not*I* for L472E, N473E, N479V, D480N, Y491F, and RBSM, respectively). The positive clones were validated by Sanger sequencing to confirm the presence of the RBSM mutation and the frame of the gene fusion within the A_1_ of Qβ. The confirmed plasmid was amplified and used for phage expression.

#### Production of recombinant phages with various RBDS1 mutants

For phage expression, 50 ng of recombinant pQβRBSM1 mutant DNA were used to transform *E. coli* HB101 and plated in 2YT agar supplemented with 100 mg/g of ampicillin (48). Two clones of the transformant were inoculated in 3 ml of 2YT supplemented with 100 mg/g of ampicillin and incubated for 5 h at 37 °C while shaking at 220 rpm. This initial culture was then transferred to 1L 2YT media supplemented with ampicillin and incubated overnight under the same conditions as the initial culture. The overnight culture was precipitated with PEG_800_/NaCl, and phages were extracted. The resulting phage solution was amplified by infecting *E. coli* Q13. The titer of the amplified phage solution was checked against *E. coli* Q13 using the agar overlay assay (48).

#### RT-PCR of recombinant phages

The RNA of recombinant RBSM mutant phages was isolated using the Qiagen Kit. The extracted RNA was reverse transcribed, and the cDNA obtained was used as a template in a PCR reaction for amplification (48). The PCR product was extracted by agarose gel electrophoresis and purified using the Qiagen gel extraction and Qiagen PCR purification kits. The purified product was submitted to Sanger sequencing to confirm the presence of the insert within the A_1_ and the presence of the mutation within the RBSM insert.

### ELISA

#### Recombinant RBDS1 mutant phages binding rhACE2

The effect of RBSM mutants to binding rhACE2 was evaluated using the ELISA Kit (Cayman Chemical). Briefly, 100 μl of recombinant phages diluted to a titer of 10^3^ pfu/ml in coating buffer (NaHCO_3_) were added to a 96-well plate and incubated at 4 °C overnight. The coating buffer was removed; the wells were washed five times with wash buffer (PBS with 0.05% Tween-20), blocked with 1% bovine serum albumin in coating buffer, and incubated at room temperature. The blocking buffer was discarded, and the wells were washed five times with wash buffer. This was followed by the addition of 100 μl of 0.625 μg/ml of rhACE2 into the wells and incubation for 1 h at room temperature on an orbital shaker. The contents of the wells were discarded, and the wells were washed five times. A volume of 100 μl of anti-His antibody (1:1000) was added to the wells and incubated for 1 h at room temperature on an orbital shaker. The contents of the wells were discarded again, the wells were washed five times, 125 μl of 3,3′,5,5′-tetramethylbenzidine (TMB) was added, and the plate was incubated for 30 min at room temperature. The reaction was stopped with 75 μl of the stop solution, and the absorbance was read immediately at 450 nm using a 96-well ELISA plate reader. All ELISA experiments were performed in triplicate with Qβ WT phage was used as a control.

#### Reactivity of recombinant phages with antibodies

The recombinant phages were diluted individually to a titration of 10^3^ pfu/ml in coating buffer (NaHCO_3_). High-binding 96-well ELISA plates were coated with the relevant diluted phages and incubated overnight at 4 °C. The wells were washed three times with PBST (PBS with 0.05% Tween-20) to remove unbound phages, then blocked with 5% bovine serum albumin and incubated at 37 °C for 1 h. The coating buffer was removed, and the plates were washed three times with PBST. The coated plates were then probed with the graded dosages of relevant monoclonal antibodies diluted in PBS at concentrations of 100, 10, 1, 0.1, and 0.01 ng/ml. A volume of 100 μl of diluted antibodies were added to each well and incubated for 2 h at 37 °C. Unbound antibodies were removed by washing the wells five times with PBST. The reactivity of the monoclonal antibodies with the recombinant phages was probed with HRP-conjugated goat anti-mouse IgG diluted at 1:5000 in 1X PBS. The bound conjugate was detected using the TMB substrate. The HRP reaction was stopped by adding 100 μl of the stop solution. The reactivity was assessed by reading the colorimetric signal at an absorbance of 450 nm on an ELISA plate reader.

#### Dot-blotting analysis

For the dot blotting analysis, 10 μl of 10^5^ pfu/ml of recombinant phages were spotted on a nitrocellulose membrane, allowed to dry for 10 to 15 min, and then blocked with a 1X roti block for 30 min. After blocking, the membrane was probed with anti-RBD rabbit mAb at a dilution of 1:1000 and incubated at room temperature for 30 min and then washed 3 × 5 min with TBST (TBS with 0.06% Tween 20). The membrane was incubated with goat anti-rabbit IgG HRP diluted 1:5000 in 1X roti block for 30 min, washed 5X with wash buffer, and the bound conjugated protein was detected by exposing the membrane to chemiluminescence. The image was taken using Odyssey LI-COR Acquisition v1.2.0.72 software.

#### Cesium chloride (CsCl) gradient purification of phage

Phage suspension at 1 x 10^9^ pfu/ml was obtained as described elsewhere (48). The phage suspension was mixed with solid CsCl to a final density of 1.40 to 1.45 g/ml by slowly adding CsCl at a rate of 0.75 g/ml of phage suspension while gently swirling to dissolve. The phage-CsCl solution was loaded into ultracentrifuge tubes and carefully balanced to within 10 mg before centrifugation. Ultracentrifugation was then run at 90,000 rpm at 15 °C for 12 h in a Beckman NVT 90. Following centrifugation, a distinct gray-white phage band was visible within the middle third of the tube. The tube was clamped onto a retort stand, and a sterile 10 cc syringe with an 18 to 20 ga. needle was carefully inserted through the tube wall, 2 to 3 mm below the band. This band containing the phage was carefully drawn out and transferred into a dialysis cassette. Dialysis was performed overnight at 4 °C against 1 L of gelatin-free SM buffer containing 1M NaCl. The dialysis buffer was replaced with a fresh gelatine-free SM buffer and dialysis was continued for an additional 2 to 3 h at room temperature. This step was repeated once to ensure complete removal of CsCl. The final purified phage solution was filter-sterilized using a 0.22 μm membrane, and its titer was determined before storage at 4 °C for subsequent cryo-electron microscopy (cryo-EM) analysis.

#### Electron microscopy analysis of purified phages

Recombinant phage morphology was verified using both negative-stain electron microscopy (EM) and cryo-EM. For EM, 5 μl of purified phages were loaded onto a carbon-coated formvar grid a concentration of 10^7^ pfu/ml, as previously described (47). The grid was allowed to air-dry briefly before staining with a few drops of aqueous uranyl acetate. Excess stain was removed, and the sample was examined using a JEOL 1200EX electron microscope.

For Cryo-EM analysis, 3 μl of CsCl purified phages was loaded onto a glow-discharged grid and blotted using a Vitrobot Mark IV. The grid was frozen in liquid nitrogen and then transferred to a transmission electron microscope. Imaging was performed at 300 kV, and micrographs were captured at 590,00x magnification according to a slightly modified protocol of Liu (56).

#### Competitive inhibition assay

The WT of SARS-CoV pseudovirus was used for the assay. The test was performed using the kit provided by Virongy Biosciences and following the manufacturer's instruction. Twenty microliters (20 μl) of recombinant phages at a titer of 10^7^ pfu/ml were tested, and the assay was performed in triplicate on three different occasions. WT phages were used as the negative control.

#### Cytotoxicity of the recombinant phages displaying SARS-CoV spike receptor-binding domain

The recombinant phages were evaluated for their effect on cell viability using HEK293T cells. The test was performed using MTT (3-(4,5-dimethylthiazole-2yl)-2,5-dimethyltetrazolium bromide) assay, as described by Vaucher *et al.* (57). Briefly, SARS-CoV host cells HEK293T (Virongy Biosciences) were prepared and added in a 96-well plate according to the kit manufacturer's instructions. Twenty microliters of each recombinant phage at 10^7^ pfu/ml was added to separate wells. Wells containing only cells served as control. Phage buffer and WT Qβ phages were used as negative controls while wells containing 0.5 μM of SDS were used as the positive control. The plate was incubated at 37 °C for 24 h in a CO_2_-incubator with 5% relative humidity. Thereafter, the culture medium was gently removed by pipetting ensuring minimal disturbance to the cells. Fifty microliters of MTT solution prepared in culture medium was added and the plate was incubated for 4 h under the same conditions as above. Subsequently, the MTT solution was gently removed without disturbing the cells, and 100 μl of dimethyl sulfoxide was added to the wells, and the plate was gently shaken for 1 min at room temperature to dissolve the formazan crystals. The absorbance (*A*) was measured at 540 nm using a microplate reader, and the cell viability was calculated (57). The test was performed in triplicate.

## Data availability

Data supporting the reported results can be found at Addgene (https://www.addgene.org, accessed on 26 July 2024) and for depositing plasmids at Addgene our Deposit Number are: 84639.

## Supporting information

This article contains supporting information.

## Conflict of interest

A. B. W., M. M. G., A. N., and A. D. are employees of Indiana University School of Medicine, which is a for-profit organization, and which hosted and supported this research project. The other authors declare that they have no conflicts of interest with the contents of this article.
