# Supplementary material for: Potential blocker of SARS-CoV entry and a narrow functionality of its spike protein motifs on Qubevirus platform
Source: J Biol Chem. 2025 Jun 12;301(7):110371. doi: 10.1016/j.jbc.2025.110371 (PMC12274811; doi:10.1016/j.jbc.2025.110371)
Supplement: Supplementary Material [file mmc1.docx]

**Supplementary Figures:**

**Supplementary Figure 1**

**Figure S1:** **Agarose gel electropherogram of the fusion PCR products of RBSM Panel A:** PCR products of the RBD mutants fused to the A_1_ gene of Qβ and amplified (~800 bp); M_1_ and M_2_ are the 10 kb and 100 bp ladders, respectively. RBSM is the WT RBD of SARS-CoV fused with A_1_, RBSMmt is the engineered RBSM motif bearing all five mutations fused to A_1_, and Ad2 is the deleted A1 gene amplified as a control without the C-terminus 150 bp gene.

**Supplementary Figure 2:**


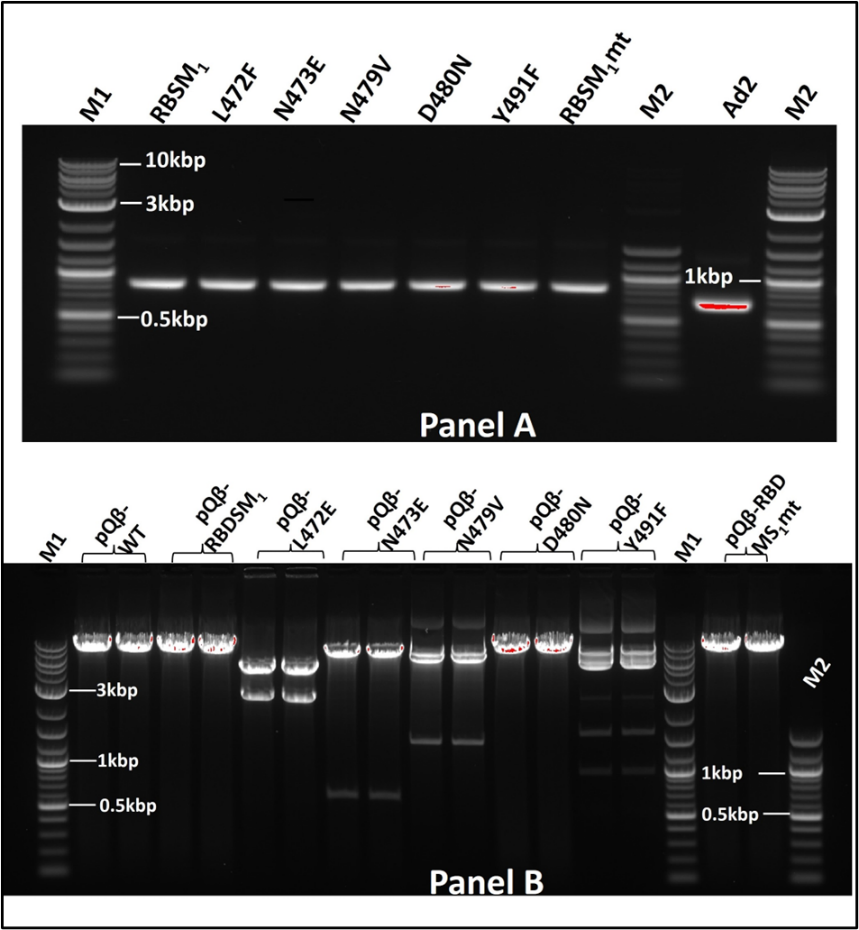


**Figure S2: Construction of recombinant RBSM mutant Qβ plasmids**. Agarose gel analysis image of the recombinant plasmid construction of RBSM mutants constructed into the backbone of the Qβ vector for RNA phage display. pQβ-WT, pQβ-RBSM, pQβ-D480N, and pQβ-RBSM_1_ are positive clones linearized with NotI; pQβ-L472E digested with NheI into 1 and 6.7 kbp fragments; pQβ-N473V digested with EcoRV into 7 kbp and 700 bp; pQβ-N479V digested into 6.5 kpb and 1.2 kpb; and pQβ-Y491F digested into 4.4, 2.3, and 1 kpb by NdeI. M_1_ and M_2_ are the 10 kb and 100 bp ladders, respectively.

**Supplementary Figure 3:**


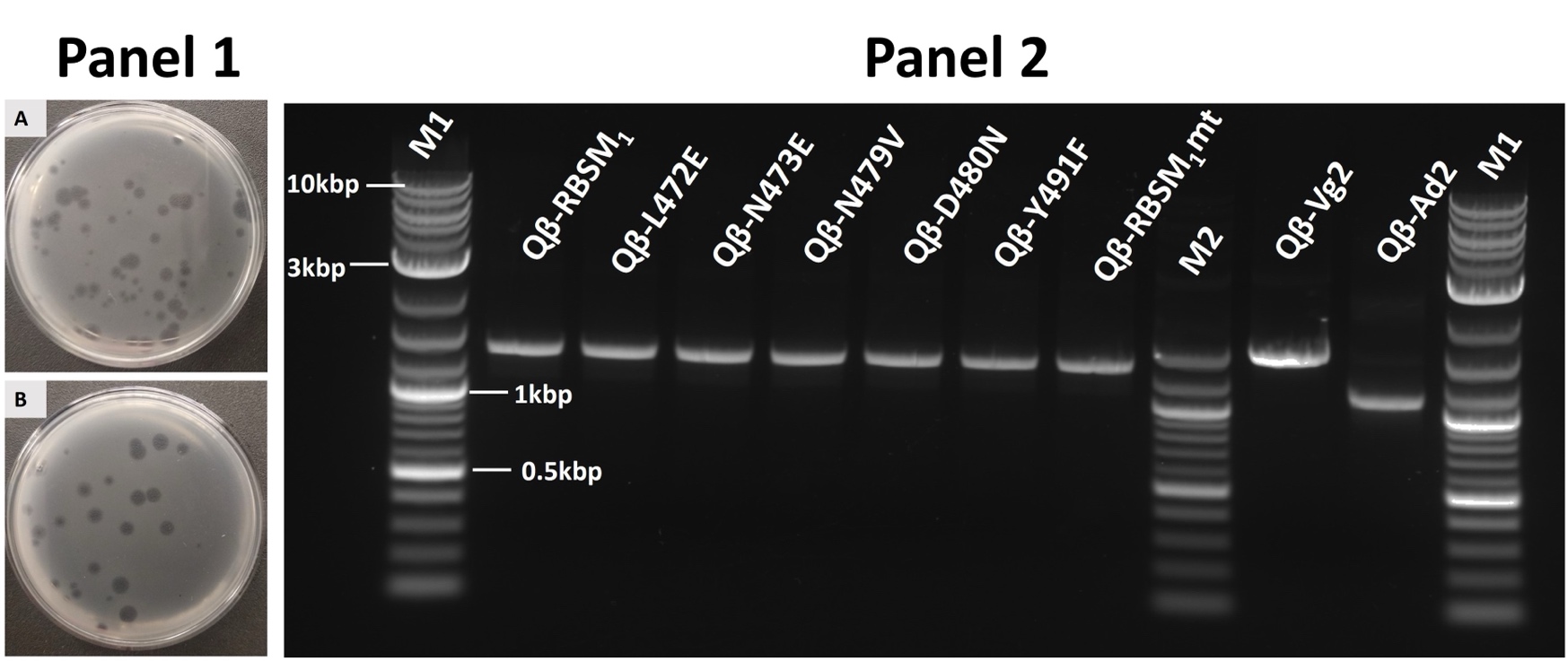


**Figure S3:** Phages Qβ-RBSM_1,_ Qβ-L472E, Qβ-N473E, Qβ-N479V, Qβ-D480N, Qβ-Y491F, and Qβ-RBSM_1_mt represent the genomic portion of the recombinant Qβ-phages displaying the respective RBSM mutant motifs amplified by RT-PCR. Qβ-Vg2 and Qβ-Ad2 represent the positive and negative controls, respectively for the corresponding genomic portion for Vg2, and the A_1_ deletion was amplified by RT-PCR and served as controls. M_1_ and M_2_ are the 10 kb and 100 bp ladders, respectively.

**Supplementary Figure 4:**

**Figure S4:** Effects of display and mutation on protein foldability. (A): The 3D structure of the minor coat A1 protein of Qβ (Qβ A1, depicted as a green ribbon. (B): The 3D structure of the RBSM (hot pink ribbon) with five key amino acids shown as yellow sticks. (C): The 3D structure of Qβ A1 (green ribbon) in fusion with the RBSM (pink ribbon) with key amino acids (yellow sticks); the foldability of both A1 and RBSM is not significantly altered. (D): The 3D structure of Qβ A1 (green ribbon) displaying the RBSM (pink ribbon) with mutated key amino acids (yellow sticks); the mutation does not affect protein folding.
